# Effector bottleneck: microbial reprogramming of parasitized host cell transcription by epigenetic remodeling of chromatin structure

**DOI:** 10.3389/fgene.2014.00274

**Published:** 2014-08-14

**Authors:** Sara H. Sinclair, Kristen E. Rennoll-Bankert, J. S. Dumler

**Affiliations:** ^1^Graduate Program in Cellular and Molecular Medicine, The Johns Hopkins University School of MedicineBaltimore, MD, USA; ^2^Department of Microbiology and Immunology, School of Medicine, University of Maryland BaltimoreBaltimore, MD, USA; ^3^Department of Pathology, The Johns Hopkins University School of MedicineBaltimore, MD, USA; ^4^Department of Pathology, School of Medicine, University of Maryland BaltimoreBaltimore, MD, USA

**Keywords:** epigenetics, nucleomodulin, DNA methylation, histone deacetylase, chromatin, *Anaplasma phagocytophilum*

## Abstract

Obligate intracellular pathogenic bacteria evolved to manipulate their host cells with a limited range of proteins constrained by their compact genomes. The harsh environment of a phagocytic defense cell is one that challenges the majority of commensal and pathogenic bacteria; yet, these are the obligatory vertebrate homes for important pathogenic species in the *Anaplasmataceae* family. Survival requires that the parasite fundamentally alter the native functions of the cell to allow its entry, intracellular replication, and transmission to a hematophagous arthropod. The small genomic repertoires encode several eukaryotic-like proteins, including ankyrin A (AnkA) of *Anaplasma phagocytophilum* and Ank200 and tandem-repeat containing proteins of *Ehrlichia chaffeensis* that localize to the host cell nucleus and directly bind DNA. As a model, *A. phagocytophilum* AnkA appears to directly alter host cell gene expression by recruiting chromatin modifying enzymes such as histone deacetylases and methyltransferases or by acting directly on transcription in *cis*. While *cis* binding could feasibly alter limited ranges of genes and cellular functions, the complex and dramatic alterations in transcription observed with infection are difficult to explain on the basis of individually targeted genes. We hypothesize that nucleomodulins can act broadly, even genome-wide, to affect entire chromosomal neighborhoods and topologically associating chromatin domains by recruiting chromatin remodeling complexes or by altering the folding patterns of chromatin that bring distant regulatory regions together to coordinate control of transcriptional reprogramming. This review focuses on the *A. phagocytophilum* nucleomodulin AnkA, how it impacts host cell transcriptional responses, and current investigations that seek to determine how these multifunctional eukaryotic-like proteins facilitate epigenetic alterations and cellular reprogramming at the chromosomal level.

## INTRODUCTION

In order to infect mammalian hosts, bacterial pathogens evolved an array of mechanisms that serve to create an environment conducive for survival, replication, and spread. While many bacterial species survive in an extracellular environment, intracellular pathogens must be capable of both entering their host cells undetected and altering the cellular milieu in order to replicate. Traditionally, the ability of bacterial-derived proteins to induce disruptions of cell signaling or major cellular processes such as NF-κB, MAPK, and JAK/STAT pathways, are the predominant focus of host–pathogen interaction studies (Brodsky and Medzhitov, 2009). Recently, there has been an increasing interest in the ability of these intracellular pathogens to direct alterations in host cell gene expression that promote survival and replication (Paschos and Allday, 2010; Bierne et al., 2012; Silmon de Monerri and Kim, 2014). It is now well recognized that bacterial pathogens can reprogram host gene expression either directly or indirectly, by altering the accessibility of gene promoters via epigenetic modifications.

Eukaryotic DNA is highly organized and gene expression tightly regulated by an orchestrated network of proteins, RNAs, and other modulators. Histone octamers, or nucleosomes, organize DNA by acting as a bobbin on which the DNA winds. The charge of the histone proteins can be covalently modified in order to more tightly or loosely associate with DNA. Histone acetylation, which imparts a negative charge, is predominantly associated with an open configuration where promoters are easily accessed by RNA polymerases. Histone methylation or phosphorylation which impart a positive charge, cause DNA to more tightly associate with histone proteins, reducing promoter accessibility to transcription activating machinery. The process of modulating open and closed chromatin is further complicated by (i) the residue(s) of which histone protein(s) is/are modified, (ii) methylation of cytosine residues in DNA, and (iii) non-coding RNAs, and by the manner in which these mechanisms are intertwined. It therefore is no surprise that bacterial-derived proteins have evolved to interfere with host gene expression that improves bacterial fitness.

Over the last decade, examples of secreted bacterial effector proteins, ranging from those produced by *Listeria monocytogenes*,* Chlamydia trachomatis*,* Shigella flexneri*, and others, were found to target the host cell nucleus (Paschos and Allday, 2010; Bierne et al., 2012; Silmon de Monerri and Kim, 2014). These discoveries demonstrate the relationship of altered transcription and function of infected host cells and microbial survival, and in some cases, pathogenicity. *S. flexneri*, a bacterial pathogen that can cause dysentery, prevents NF-κB from binding its target gene promoters by altering the phosphorylation state of histone H3 at serine 10. The bacterium does so by secreting outer membrane protein F (OspF) that de-phosphorylates MAPKs in the nucleus resulting in a lack of histone H3 phosphorylation at serine 10 at a number of NF-κB-dependent genes (Arbibe et al., 2007). By altering NF-κB target gene transcription, *S. flexneri* suppresses the host cell inflammatory response promoting microbial survival and transmission (Arbibe et al., 2007). *L. monocytogenes* nuclear targeted protein A (LntA), blocks binding of heterochromatin inducing protein BAHD1 at interferon-stimulated genes resulting in upregulated expression (Rohde, 2011). Nuclear effector E (NUE) of *C. trachomatis* and RomA of *L. pneumophila* have methyltransferase activity and induce methylation of eukaryotic histones and altered host cell gene expression (Pennini et al., 2010; Rolando et al., 2013). Plant pathogens in the genus *Xanthomonas*, as well as *Ralstonia solanacearum*, *Burkholderia rhizoxinica*, and an endosymbiont of the plant pathogenic fungus *Rhizopus microsporus* all possess genes encoding transcription activator-like (TAL) protein effectors that when expressed, bind plant DNA and alter transcription and disease susceptibility (Sugio et al., 2007; Bogdanove, 2014). These examples are part of an expanding array of bacterial-derived proteins termed nucleomodulins that target host cell chromatin or chromatin-linked pathways to alter transcription, typically at one or a few host genes. To date, the only prokaryotic nucleomodulins shown to directly bind mammalian DNA and influence surrounding chromatin are from the *Anaplasmataceae* family. Ankyrin A (AnkA) of *Anaplasma phagocytophilum*, as well as Ank200 and several tandem-repeat containing proteins (TRPs) from *Ehrlichia chaffeensis*, have been shown to enter the nucleus and bind DNA, and interact with host epigenetic machinery or alter nearby histone octamers (Garcia-Garcia et al., 2009a; Luo et al., 2011; Zhu et al., 2011; Luo and McBride, 2012; Dunphy et al., 2013).

Targeting of individual genes or binding of an effector at a single chromatin region or small numbers of promoter loci could, in theory, lead to *cis* regulation of those loci and in part explain transcriptional alterations induced by infection. However, the degree of transcriptional alterations and the coordination of these events that dramatically affect cellular function programs are unlikely to be explained by individual targets given the complexity and repertoire of the human genome, at approximately 3251 Mb vs. genome sizes of *S. flexneri* (≤4.83 Mb; Jin et al., 2002), *C. trachomatis* (≤1.04 Mb; Stephens et al., 1998), *L. monocytogenes* (≤3.00 Mb; Evans et al., 2004), *L. pneumophila* (≤3.40 Mb; Chien et al., 2004), and *A. phagocytophilum* (≤1.47 Mb; Lin et al., 2011). We hypothesize that nucleomodulins can act broadly, even genome-wide, to affect entire chromosomal neighborhoods and topologically associating chromatin domains by recruiting chromatin remodeling complexes or by altering the folding patterns of chromatin that bring distant regulatory regions together to coordinate control of transcriptional reprogramming. This review will discuss the current knowledge of *A. phagocytophilum* subversion of host cells by nucleomodulins and how AnkA could play an even larger role than its documented effects on transcription.

## Anaplasma phagocytophilum

*Anaplasma phagocytophilum,* transmitted by *Ixodes* spp. ticks, was discovered as the causative agent of human granulocytic anaplasmosis (HGA) in 1990 (Chen et al., 1994). While the infection is usually subclinical, manifestations in humans range from mild fever to severe infection requiring intensive care or even death (Chen et al., 1994). *A. phagocytophilum* is a Gram negative, obligate intracellular bacterium that is a parasite of neutrophils in mammalian hosts. Owing to their roles in protective inflammatory and immune responses toward microbial infections and their innate ability to recognize and kill pathogens, neutrophils are unlikely host cells for any microorganism. Yet, *A. phagocytophilum* colonizes these cells and thwarts their normal functions to create a hospitable environment for intracellular replication and subsequent transmission via tick bite. With a limited genome of approximately 1.5 Mb (Lin et al., 2011) the potential genetic reservoir for controlling an infected host cell, whether in the tick or mammal, is only a fraction of the tick or human genome. This simple observation suggests that to circumvent such extremes, its control mechanisms must be highly efficient, multifunctional, or target master regulators or similar checkpoints in eukaryotic cells.

One remarkable feature of *A. phagocytophilum*-infected cells is the marked change in transcriptional profiles that belies aberrant regulation of many key host pathways (Borjesson et al., 2005; de la Fuente et al., 2005; Lee et al., 2008). *A. phagocytophilum*-infected neutrophils are characterized by an “activated-deactivated” phenotype with major functional aberrations including decreased respiratory burst, delayed apoptosis, reduced transmigration across endothelial cell barriers, and decreased antimicrobial activities such as phagocytosis and microbial killing, while simultaneous increases in degranulation of vesicle contents including proteases and increased production of chemokines, are observed (Carlyon et al., 2002; Carlyon and Fikrig, 2003; Choi et al., 2005; Dumler et al., 2005; Garyu et al., 2005; Ge et al., 2005; Carlyon and Fikrig, 2006; Garcia-Garcia et al., 2009b). The net result of these changes is an increase in (i) inflammatory responses that recruit new host cells, (ii) prolonged survival of infected cells, (iii) an inability to kill internalized microbes, and (iv) net sequestration of infected cells within the intravascular compartment that is more readily accessed by the next tick bite (Rennoll-Bankert and Dumler, 2012). In fact, interference with any of these processes using *in vitro* and *in vivo* models leads to reduced fitness of the microbe underscoring how important these functional changes are in mammalian hosts.

The biological basis for such dramatic changes in neutrophil function is increasingly studied by methods that range from examination of individual pathways to genome-wide systems biology approaches. Transcriptional profiling of infected neutrophils and HL-60 cells, the latter a commonly used cell model for *A. phagocytophilum* infection, reveals altered transcription genome-wide, confirming that changes in transcription are not restricted to a few genes and limited cellular functions, but likely play a role in most of the known functional changes induced by infection (Borjesson et al., 2005; de la Fuente et al., 2005; Lee et al., 2008). Transcriptional downregulation of two components of the NADPH oxidase, *CYBB* encoding NOX2 or gp91^phox^ and the GTPase* RAC2*, that assemble in the membranes of activated cells to generate superoxide production and promote microbial killing plays a role in prolonged inhibition of respiratory burst (Banerjee et al., 2000; Carlyon et al., 2002). In addition, delayed apoptosis is achieved by maintained transcription of *BCL2* family members (Choi et al., 2005; Ge et al., 2005) and increased proinflammatory responses are due to upregulated transcription of cytokine and chemokine genes such as *IL8* (Klein et al., 2000; Akkoyunlu et al., 2001; Scorpio et al., 2004).

While many studies document these changes at functional and transcriptional levels, the precise mechanisms that organize and coordinate alterations to support improved *A. phagocytophilum* fitness are not well addressed. The biggest advance in understanding these processes occurred with the discovery of secreted prokaryotic effector proteins and their secretion systems. The genome of *A. phagocytophilum* encodes a type IV secretion system (T4SS) that allows protein effectors to translocate into infected host cells where they likely act by mechanisms similar to those of other bacterial secreted effectors that target cytosolic pathways such as signal transduction, cytoskeletal rearrangements, intracellular trafficking, etc. (Ohashi et al., 2002; IJdo et al., 2007; Gillespie et al., 2009). These observations also hold true for *A. phagocytophilum* effectors. However, not all effector proteins remain localized within the host cytosol, and those that enter the nucleus have direct access to genes as well as to a distinct and diverse array of proteins that could impact cellular function on a global scale.

## AnkA OF *Anaplasma phagocytophilum*

*Anaplasma phagocytophilum* expresses a number of major immunoreactive proteins, including major surface protein 2/p44 (Msp2/p44) and AnkA, the latter of which was found by Caturegli et al. (2000) using immunoelectron microscopy to be transported into the host cell nucleus and bound within heterochromatin (Park et al., 2004). AnkA contains many EPIYA motifs that become tyrosine phosphorylated and recruit SHP-1 and ABL1 when introduced into mammalian cells which in turn regulates endosomal entry and intracellular infection (IJdo et al., 2007; Lin et al., 2011). Aside from this, AnkA contains multiple eukaryotic motifs including 8–15 or more ankyrin repeats, a putative bipartite nuclear localization signal (NLS), and a putative high mobility group N-chromatin unfolding domain (HMGN-CHUD; Caturegli et al., 2000). The ankyrin repeat domains are organized tandemly, creating highly stable spring-like structures that allow protein–protein or protein–DNA interactions and are commonly found in transcription factors and their regulatory proteins (Bork, 1993; Jernigan and Bordenstein, 2014). HMGN-CHUD domains facilitate binding to nucleosomes to alter chromatin structure and transcription of surrounding genes (Bustin, 1999). The presence of these motifs lends credence to the idea that AnkA plays a role in altering neutrophil transcription.

Park et al. (2004) confirmed that AnkA directly binds both DNA and nuclear proteins, and provided limited evidence of its capacity to bind broadly throughout the human genome. AnkA binds to regions at the promoter of *CYBB*, encoding the gp91^phox^ component of phagocyte oxidase (Garcia-Garcia et al., 2009b). *CYBB* is known to be transcriptionally repressed with *A. phagocytophilum* infection, further suggesting that AnkA acts in *cis* to alter gene transcription (Thomas et al., 2005; Garcia-Garcia et al., 2009b). Moreover, transcription of *CYBB* is decreased in a dose dependent manner as nuclear AnkA concentrations increase (Garcia-Garcia et al., 2009b). When AnkA-expressing plasmids are transfected into HL-60 cells, *CYBB* expression is dampened, strengthening the link to transcriptional regulation (Garcia-Garcia et al., 2009b). In electrophoretic mobility shift assays, AnkA binds to the *CYBB* promoter at the same locations as other known transcriptional regulators such as CAATT displacement protein (CDP) and special-AT rich binding protein-1 (SATB1; Thomas et al., 2005; Garcia-Garcia et al., 2009b; Rennoll-Bankert and Dumler, 2012). Surprisingly, DNA binding by AnkA does not target a conserved DNA sequence motif or signature; rather it binds to regions rich in AT nucleotides that have specific structural qualities, including the ability to uncoil under superhelical stress (Park et al., 2004; Garcia-Garcia et al., 2009b). The latter feature is often observed in eukaryotic proteins that tether DNA strands together into the nuclear matrix at matrix attachment regions (MARs) to regulate transcription from distantly located but functionally related chromosomal regions; proteins that bind these regions are called MAR-binding proteins (MARBPs).

Interestingly, Garcia-Garcia et al. (2009a) showed that multiple downregulated defense genes in infected granulocytes were clustered on chromosomes. The spatial clustering of similarly regulated genes suggests that, in addition to *cis*-regulation as with *CYBB*, long ranges of chromosomes are affected – a process often attributed to epigenetic mechanisms such as DNA methylation and histone chromatin modifications. Using chromatin immunoprecipitation (ChIP) to investigate histone marks at defense gene promoters, acetylation of histone H3 was dramatically reduced, a finding often associated with silenced gene transcription (Garcia-Garcia et al., 2009a). To explain this, binding of histone deacetylase-1 (HDAC1) was found increased across multiple defense gene promoters (Garcia-Garcia et al., 2009a). Moreover, *A. phagocytophilum*-infected granulocytes have increased HDAC activity most likely explained by the increased quantity of both HDAC1 and HDAC2. Inhibition of *HDAC1*, but not *HDAC2* expression by siRNA or pharmacologic inhibition of HDAC activity impairs *A. phagocytophilum* propagation, whereas overexpression leads to increased intracellular propagation (Garcia-Garcia et al., 2009a). We modeled this process by using the wild type *CYBB* promoter, to which AnkA binds, and mutated forms unable to bind AnkA in order to demonstrate that AnkA binding leads to HDAC1 recruitment and silencing of expression at *CYBB* (Garcia-Garcia et al., 2009b). These results clearly link AnkA with HDAC1 toward facilitating widespread downregulation of antimicrobial responses.

Currently, most data examining the ability of HDAC1 and AnkA to modulate transcription focuses on a small number of loci, including *CYBB* and up to 17 other gene promoters. While AnkA and HDAC1 are important contributors to transcriptional downregulation of some genes in *A. phagocytophilum-*infected cells, neither has been determined to provide a broad mechanism that coordinates the transcriptional or functional alterations observed. Importantly, transcriptional profiling of *A. phagocytophilum*-infected granulocytes, including both primary neutrophils and cell lines, demonstrates that the majority of differentially expressed genes are upregulated (Borjesson et al., 2005). Thus, while HDAC1 recruitment by AnkA is important for down-regulating some genes, the majority of DEGs are likely regulated by alternative or additional mechanisms.

**FIGURE 1:**
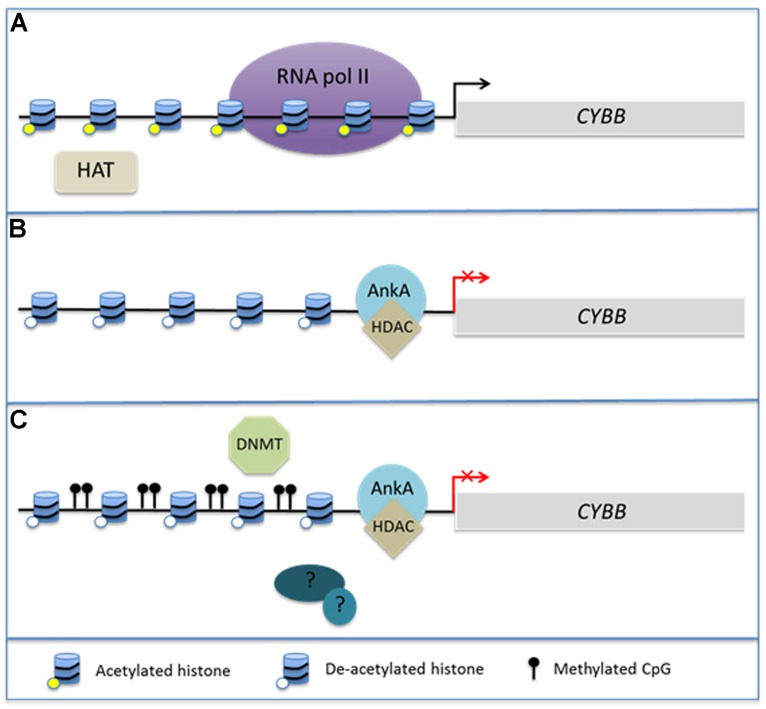
**AnkA alters chromatin structure at the *CYBB* promoter.** During *Anaplasma phagocytophilum* infection, AnkA accumulates in the host cell nucleus where it can directly bind DNA and influence the transcription of *CYBB*, which encodes the gp91^phox^ component of the NADPH oxidase. **(A)** In the absence of infection, *CYBB* is activated by inflammatory signals and is easily transcribed. **(B)** During infection, AnkA binds the proximal promoter and recruits HDAC which deacetylates nearby histones in order to inhibit transcription. **(C)** We propose that additional host or bacterial-derived chromatin modifying enzymes, such as DNMT methylation of host CpGs, may also be involved in altering the host epigenome to the bacterium’s advantage.

Of considerable interest is the interplay between chromatin structure and DNA methylation. Histone modifications, DNA methylation at CpG islands, and binding of methyl CpG DNA-binding proteins (MeCPs) via their methyl-DNA binding domains (MBDs) occur in concert during biological responses including neoplasia and cellular differentiation (Baylin and Herman, 2000; Baylin et al., 2001; Stirzaker et al., 2004; Srivastava et al., 2010; Reddington et al., 2013). Cytosine residues of CpG dinucleotides can by methylated by DNA methyltransferases (DNMTs) leading to sustained DNA methylation over multiple generations of cell divisions. It is believed in part that the methylation protects genes and transcriptional programs from inappropriate ectopic expression as cells enter various stages of tissue differentiation (Srivastava et al., 2010). DNMT1 is highly conserved and is thought to be primarily responsible for maintaining existing methyl CpGs (Baylin et al., 2001). In contrast, DNMT3a and 3b are believed to undergo transient *de novo* expression that induces CpG methylation in response to cellular stimuli (Okano et al., 1999). MBDs can recognize these newly methylated regions of DNA and are often associated with HDACs in MeCP1 and MeCP2 complexes, illustrating the potential for a direct link between DNA methylation and alterations in chromatin structure (Jones et al., 1998; Nan et al., 1998; Ng et al., 1999). Generally, hypermethylation of gene promoters occurs synchronously with histone deacetylation and decreased gene expression of many tumor suppressor genes (Baylin and Herman, 2000; Baylin et al., 2001; Stirzaker et al., 2004). This complex interplay has been best studied in cancer genomes, where it is currently unclear whether DNA methylation induces alterations in chromatin structure or if chromatin structure induces changes in DNA methylation. However, given the interplay between HDAC activity and DNA methylation, it is not unreasonable to hypothesize that *A. phagocytophilum* infection induces hypermethylation of the host genome during the process of global transcriptome reprogramming (**Figure 1**).

In addition to chromatin alterations induced by HDACs, MARs are responsible for dictating the three-dimensional architecture of chromatin loops and serve as tethering points for DNA to the nuclear matrix (Spector, 2003; Kumar et al., 2007; Kohwi-Shigematsu et al., 2012). Arranging chromatin into loops allows transcription factors attached to the matrix access to promoters and brings distal genomic loci and regulatory regions into a position of proximity for coordinated regulation (Arope et al., 2013). Interestingly, the AT-rich DNA docking sites of AnkA are similar to those of MARs. In fact, several transcription factors including SATB1, bind MARs to coordinately modulate transcription from large genomic regions (Kumar et al., 2007). Like AnkA, SATB1 occupies the *CYBB* promoter during myeloid differentiation where it represses the transcription of *CYBB*, is involved in maintaining expression of *BCL2* (Gong et al., 2010) which is transcriptionally sustained during *A. phagocytophilum* infection (Ge et al., 2005) and is implicated in activation of cytokine expression in T cells (Hawkins et al., 2001; Beyer et al., 2011). SATB1 interactions with chromatin remodeling complexes include HDACs which are likely involved in some of these processes (Yasui et al., 2002). The complex looping of chromatin facilitated by MARs and MAR binding proteins allows for entire chromatin domains and territories to be remodeled despite relatively few binding sites (Cai et al., 2006). For example, SATB1 binds to only nine regions across the 200 kb T_H_2 locus, yet it is a major regulator of T_H_2 lymphocyte differentiation and function (Cai et al., 2006). Complex protein interactions involving chromatin remodeling and histone modification mediated by anchoring proteins like SATB1 make it plausible that DNA-binding bacterial nucleomodulins such as AnkA could target broad transcriptional programs that belie functions of host cells. While only limited data currently exist, AnkA binds to at least 23 distinct sites on 12 separate chromosomes with *A. phagocytophilum* infection of the human HL-60 promyelocytic cell line (Park et al., 2004) detailed mapping of AnkA genomic binding sites is now in progress. We therefore think it is plausible that AnkA binds throughout the genome and exerts its effects, like SATB1, to both repress and activate transcription by tethering chromatin to the nuclear matrix and exposing promoters to chromatin remodeling complexes.

## *Anaplasma phagocytophilum* INFECTION IS ASSOCIATED WITH TRANSCRIPTIONAL CHANGES OVER MEGABASES IN THE HUMAN GENOME

As a relatively new field, studies that examine the epigenome of cells infected by or as a consequence of bacterial infection, whether parasitic or symbiotic, are few and far between. Given the proven interaction of *A. phagocytophilum* AnkA with gene promoters, epigenetic alterations of nearby chromatin, and its MAR-binding attributes, we reanalyzed publicly available transcription microarray data generated from *A. phagocytophilum*-infected human peripheral blood neutrophils (Borjesson et al., 2005). We used updated bioinformatics tools, including an analysis of the SD estimates for differential transcription at each locus, and wherever possible, improved gene and gene feature localization using the NCBI GRCh38 human genome assembly (release date December 24, 2013). The differential expression of all 18,400 interrogated transcripts and variants, including 14,500 well-characterized human genes, was mapped by chromosomal position so that the relationship between the linear chromosome landscape and transcriptional activity could be visualized and investigated. To display physical locations on chromosomes with long regions of altered transcription, the fold change of expression for each locus was averaged with the nearest neighboring genes to create sliding windows for each chromosome. Overall, the median sliding window included 10.0 genes/gene features (IQR 2.0) spanning a median interval of 2.04 Mb (IQR 2.16) corresponding approximately to the lower end dimensions of chromosomal or gene expression dysregulation domains (Letourneau et al., 2014). The sliding window average along with individual gene transcriptional fold changes were plotted for each chromosome. To assess significance of fold changes in the sliding window, a similar sliding window was created to simultaneously show the average estimated SDs over each window.

**FIGURE 2:**
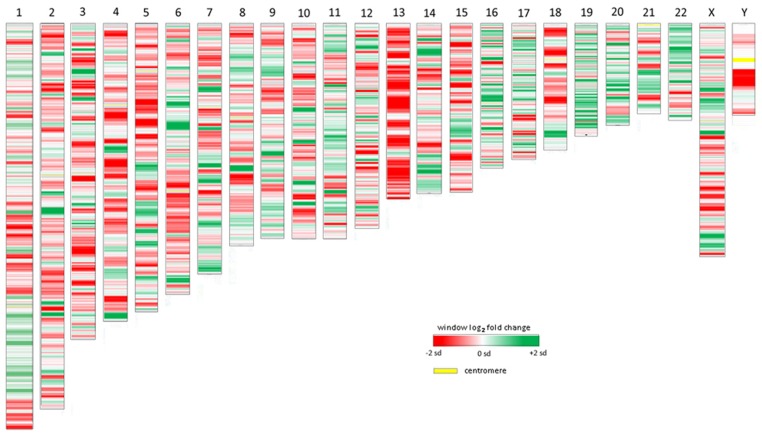
**Heat map demonstrations of long linear chromosomal differential transcription of *A. phagocytophilum*-infected human neutrophils vs. uninfected neutrophils.** The human chromosome number is shown at the top. The colors represent estimates of the SD of differential transcription of the window at each position compared to the overall differential transcription> for that chromosome; dark red = -2 SDs; dark green = +2 SDs.

**FIGURE 3:**
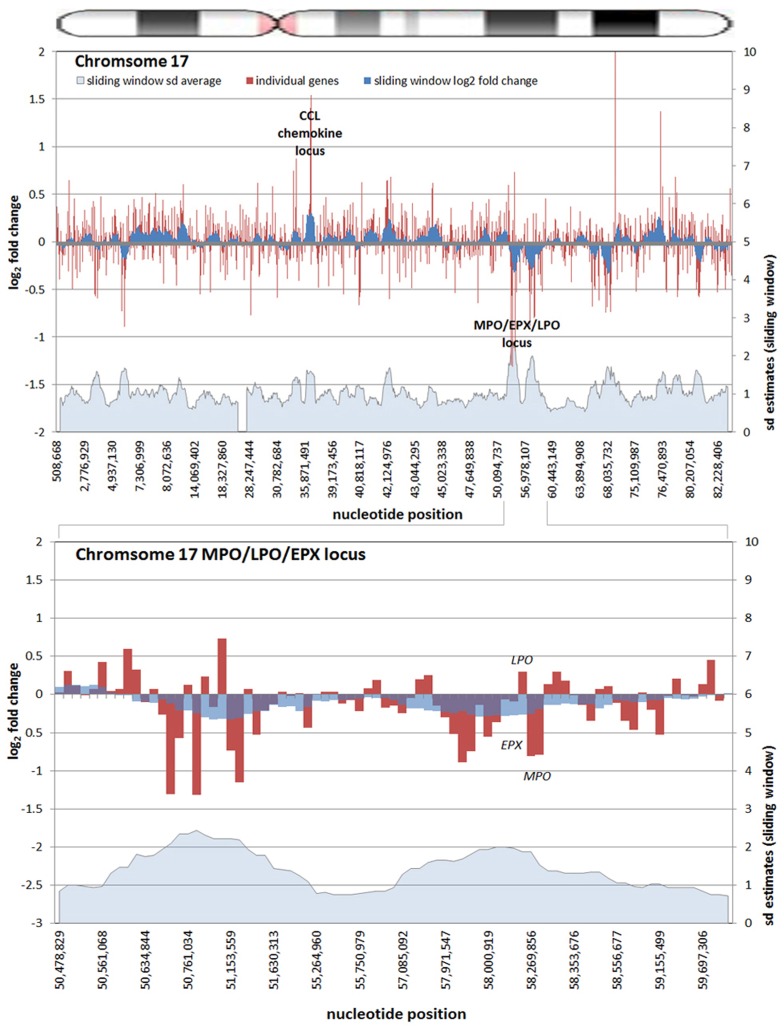
**Differential gene expression patterns cluster over large linear genomic regions in *A. phagocytophilum*-infected human peripheral blood neutrophils.** The top panel shows human chromosome 17; the bottom panel shows the* MPO*/*EPX*/*LPO* locus on human chromosome 17 and the large genomic region that is upregulated with infection. The chromosome ideogram is shown at the top. Red bars (left axes) represent differential transcription of individual genes, including some replicates; the dark blue zones (left axes) show the sliding window average log_2_-fold differential transcription over the contiguous 9–11 genes (or 0.38–1.25 Mb). The light blue zone at the bottom (right axis) shows the sliding window average over the same region for estimated SDs of log_2_-fold differential transcription at each gene or gene feature. Data re-analyzed from Borjesson et al. (2005) www.ncbi.nlm.nih.gov/geo, accession no. GSE2405.

**FIGURE 4:**
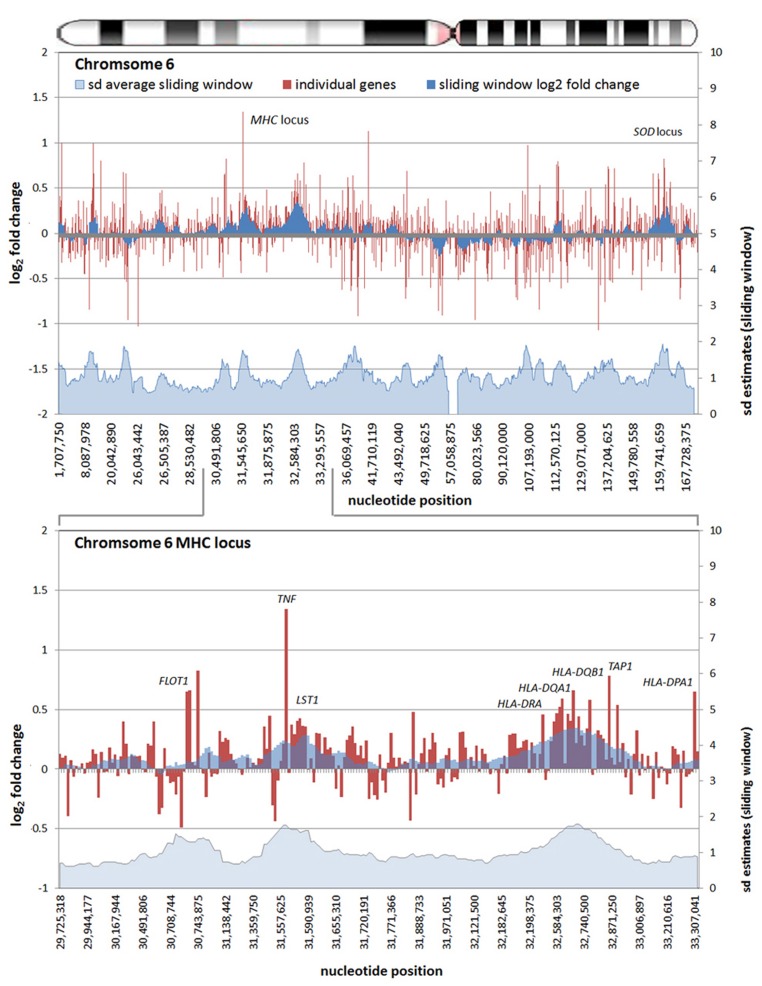
**Differential gene expression patterns comparing *A. phagocytophilum*-infected vs. non-infected human peripheral blood neutrophils are clustered over large linear genomic regions.** The top panel shows human chromosome 6; the bottom panel shows the MHC locus on human chromosome 6 including a large genomic region spanning the *TNF* and *HLAD* loci that are upregulated with infection. The chromosome ideogram is shown at the top. Red bars (left axes) represent differential transcription of individual genes, including some replicates, the dark blue zones (left axes) show the sliding window average log_2_-fold differential transcription over the contiguous 9–12 genes (or 0.42–3.24 Mb). The light blue zone at the bottom (right axis) shows the sliding window average over the same region for estimated SDs of log_2_-fold differential transcription at each gene or gene feature. Data re-analyzed from Borjesson et al. (2005) http://www.ncbi.nlm.nih.gov/geo, accession no. GSE2405.

All chromosomes showed linear regions of marked differential transcription covering megabases in scale on both p and q arms (**Figure 2**). In keeping with the original observations of Borjesson et al., most clusters were upregulated; however, the analysis also demonstrated a similar pattern of downregulated genes over long linear regions. Of interest was chromosome 17 with a 9.4 Mb cluster containing myeloid peroxidase (*MPO*), eosinophil peroxidase (*EPX*), and lactoperoxidase (*LPO*) which showed marked downregulation, and a 3.2 Mb cluster containing multiple upregulated chemokine genes (**Figure 3**). In addition to reduced transcript levels, *MPO* and *EPX* promoters were previously shown to be deacetylated upon *A. phagocytophilum* infection (Garcia-Garcia et al., 2009a). Furthermore, a 3.6 Mb cluster on chromosome 6 containing *FLOT1*, *TNF*, and the major histocompatibility complex (*MHC*) was upregulated (**Figure 4**). The chromosomal landscape of the *MHC* locus is well documented with respect to chromatin looping via MARS and histone marks (Ottaviani et al., 2008). It is difficult to determine with certainty whether these changes are the result of manipulation by microbial effectors like AnkA, or whether these represent host cellular responses to infection, or an amalgam of both. The limited genome-wide transcriptional alterations originally documented with exposure to heat-killed *A. phagocytophilum* argue for the former (Borjesson et al., 2005). Regardless, these observations clearly suggest that *A. phagocytophilum* infection targets large chromosomal territories to induce transcriptional alterations, in addition to specific genes. Given that HL-60 cells transfected to express AnkA have nearly identical differential gene expression patterns (Garcia-Garcia et al., 2009b) and reductions in induced respiratory burst compared to *A. phagocytophilum*-infected cells, a hypothesis that prokaryotic nucleomodulins evolved to modulate cell function by epigenetic alterations is compelling.

## CONCLUSION AND FUTURE DIRECTIONS

Given the limited nature of bacterial genome repertoire, and the expansive genome repertoire, organization and complexity of transcriptional regulation in eukaryotes, the ability of prokaryotes to alter eukaryotic host cell functions at the epigenetic level is truly a remarkable phenomenon. The processes by which bacteria-derived proteins alter host cell signaling, endocytic or vesicular trafficking, and* cis* transcription of genes are important. However, these pathways become challenging as paradigms that can account for the marked and diverse changes in host cell transcription and functions during the course of infection. We believe that successful intracellular prokaryotes, whether parasitic and pathogenic, mutualistic or symbiotic, evolved multifunctional and complex proteins with broad effects that target key master regulators or checkpoints that regulate major cellular reprogramming events. The *A. phagocytophilum* genome is a fraction of the size of its human host, yet it manages to disrupt core functions of the cell by transforming the transcriptome and reprogramming the cell for improved microbial fitness, survival, and transmission. It is increasingly apparent that *A. phagocytophilum* and perhaps other intracellular prokaryotes manipulate their hosts with a high degree of efficacy, but by altering the “on” or “off” state of genes in a *cis* only fashion or targeting individual or small numbers of signaling pathways is unlikely to account for this alone, in essence, an “effector bottleneck”. An analysis of available infection transcriptome data demonstrates that large chromosomal territories appear to be coordinately regulated – up or down. This observation, along with HDAC recruitment by AnkA, lends strong evidence to the idea that *A. phagocytophilum* induces global changes in host gene expression via a broad mechanism that involves epigenetic regulation of transcriptional programs which belie cellular functions.

It is currently unclear as to whether HDAC recruitment is the predominant mechanism by which AnkA exerts its chromatin modulating effects, including whether there are other host factors [e.g., polycomb repressive or hematopoietic associated factor-1 (HAF1) complexes] or additional bacterial-derived nucleomodulins that further contribute to the dramatic changes observed. To examine this, we designed a genome-level bioinformatics approach to predict nucleomodulins based on the combination of secretory and NLSs and studied the genomes of 12 phylogenetically diverse intracellular prokaryotic pathogens, including *Chlamydia pneumoniae*,* Mycobacterium tuberculosis*, and* Yersinia pestis* among others, and identified between 7 and 35 candidates for each (Borroto et al., 2009). Among these and by using iTRAQ proteomics approaches, we identified other proteins encoded in the *A. phagocytophilum* genome that localize to the host cell nucleus and are examining whether these too contribute to chromosomal structure alterations or work in synergy to enhance AnkA function (Gilmore et al., 2012). Elucidating the exact locations where AnkA or other nucleomodulins bind throughout the genome, and determining the architecture of the surrounding chromatin will be crucial to understanding this process. The interplay between HDACs, DNMTs, and methyl binding proteins suggests that *A. phagocytophilum* infection could also induce widespread DNA methylation perhaps as a mechanism for obtaining broad epigenetic changes and functional reprogramming.

Understanding the role of prokaryotic control over complex eukaryotic transcription machinery in lieu of the bottleneck that would occur with single effector targets at signaling pathways will allow for better understanding of the essential components of whole cell transcriptional re-programming. However, this has implications not only for host–pathogen or host–symbiont interactions, but across biology. The ability of proteins like AnkA of *A. phagocytophilum* and OspF of *S. flexneri* to alter inflammatory responses could provide opportunities for engineering of therapeutic agents which interfere with cellular responses in inflammatory diseases or other conditions where epigenetic factors control or contribute to disease.

## Conflict of Interest Statement

The authors declare that the research was conducted in the absence of any commercial or financial relationships that could be construed as a potential conflict of interest.
